# Supplementary material for: Pain Standards for Accredited Healthcare Organizations (ACDON Project): A Mixed Methods Study
Source: J Pers Med. 2021 Feb 5;11(2):102. doi: 10.3390/jpm11020102 (PMC7914789; doi:10.3390/jpm11020102)
Supplement: Supplementary file 1 [file jpm-11-00102-s001.zip › jpm-1063820 supplementary materials/Table S1.docx]

Table S1: Questionnaire 0 Delphi process.

During the first phase of the project, a literature review of scientific evidence has been carried out. The CORE panel expert group has identified a set of good practice standards in the management of cancer pain. These proposed standards are what we ask you to rate on a scale of 0 to 10 points (0-do not agree with the proposed standard to 10-strongly agree with the proposed standard) according to your judgement and experience to establish those standards with the highest level of consensus. It is possible that two waves (rounds of questions) will be conducted to refine these standards.

Prioritize on a scale of 0 (do not agree) to 10 points (strongly agree) your level of agreement with the proposed standards of good practice in pain management. If you wish to propose additional standards, you can suggest them in the space provided in each block of questions for this purpose in each block of questions.

| Oncology pain assessment and counseling |
| --- |
| Criteria: |
| 1. To have an agreed protocol based on the evidence that specifies context, assessment methods, classification, treatment and monitoring of how the assessment of pain in cancer patients is carried out. |
| 2. At the time of admission, a comprehensive pain assessment should be carried out and recorded in the digital medical record. |
| 3. A periodic re-evaluation of the oncological pain and its impact on the patient's quality of life should be carried out during the admission, using multidimensional scales validated and agreed as appropriate (for example DN4, NIPAC, BPI). |
| 4. The use of validated scales for the assessment of psychological well-being (e.g., HADS) should be incorporated |
| 5. The type and intensity of pain described by the patient should be systematically coded in the medical record. |
| 6. Patients should have a re-evaluation of the oncological pain (by telephone) one month after discharge from the hospital. |
| 7. The mean and range of pain scores should be monitored at the onset of pain and subsequently to measure treatment effectiveness. |
| 8. Electronic communication channels should be available to assess oncological pain and integrated care rapidly. |
| 9. The approach to patients with oncological pain should be conducted by a multidisciplinary team. |
| 10. The patient and family members (if authorized by the patient) should receive information and advice about the different types of pain (e.g., through documented patient and family-friendly printed material recommended by reliable sources). |
| 11. A support plan and health training should be carried out for family members and caregivers to prevent emotional overload of managing cancer pain. |
| 12. The patient should receive, upon request, information on patient associations specifying contact information and the functions and services they provide.  13. Information on self-help groups should be provided to those patients who meet criteria and who may benefit from this alternative for addressing pain. |
| 14. The patient should receive information on the center's portfolio of services, possible referrals to reference centers and, in general, on the care circuit for dealing with chronic oncological pain. |

| Pharmacological treatment |
| --- |
| Criteria: |
| 1. Patients suffering from oncological pain will have a continuous therapeutic guideline of treatment, including rescue treatment, to control breakthrough pain (assuring scheduled administration, at fixed times, to avoid the onset of pain). |
| 1. Preventive medication should be administered for pain relievers' unwanted effects (with particular consideration for preventing and treating opioid-induced constipation). |
| 1. The oral route will be the one chosen for the administration of therapies unless there are established contraindications. |
| 1. A specific record should be kept in the digital medical record of the "itinerary" through the WHO analgesic ladder, as well as the reasons for it (at the discretion of your responsible physician), to facilitate better coordination between the responsible team and the inter-current (emergency) teams |
| 1. The most frequent side effects of analgesic and adjuvant treatments should be monitored. |
| 1. A therapeutic plan should be established by consensus with the patient, which includes the patient's analgesic objectives (if possible quantitative employing the same scales of initial evaluation), as well as the temporality of its re-evaluation. |
| 1. An adequate analgesics pattern with different mechanisms to enhance their action (peripheral and centrally acting analgesics) should be indicated. |
| 1. There must be a plan for monitoring adherence to drug therapy. |

| Non-pharmacological treatment |
| --- |
| Criteria: |
| 1. The patient should evaluate with the professional which non-pharmacological alternatives can be added to the pharmacological treatment, for example:  - To promote rest and relaxation. - Diet adapted to the patient's situation - Adaptation of physical spaces to your needs. - Integrate the family into care. - Promoting hobbies. - An active attitude of the professionals. - Application of moderate heat/cold therapies. - Gentle massage. - Aromatherapy - Reflexology - Active and passive mobilizations - Music Therapy - Art Therapy - Meditation - Humor and Laughter Therapy - Active listening - Viewing |
| 1. Psychosocial support should be offered to the patient for better pain management, from the time of diagnosis and throughout the care process according to the patient's needs given the impact and repercussions of the disease on the patient and their social and family environment. |
| 1. A primary caregiver identification should be made for all patients with chronic pain, along with an assessment of their degree of overload. |
| 1. Spiritual and religious service will be offered to all those patients with chronic pain who request it. |
| 1. Information will be provided on the impact of drugs on body image and aesthetic strategies to reduce it. |
| 1. In the case of pain that does not subside with non-invasive treatment, a non-pharmacological interventionist technique should be proposed to the patient. |

| Palliative Care |
| --- |
| Criteria: |
| 1. According to the existing care structure in their geographical area, all patients should have access to palliative and supportive care throughout their illness. |
| 1. A consensus protocol for referral to palliative care developed with a multidisciplinary perspective should be applied. |
| 1. There should be a calendar of joint sessions of Oncology, Pain Unit and Palliative Care. |
| 1. The patient should be informed of the essential functions of the palliative care service. |
| 1. The suffering of the patient with oncological pain should be systematically assessed and accompaniment promoted. |
| 1. The accompaniment of the Home Hospitalization Units and the chronic hospitals (or HACLE type) in their work of palliative care of patients with chronic oncological pain must be verified. |

| Coordination |
| --- |
| Criteria: |
| 1. Each service must have established a care route that collects the patients' schedule's adequate coordination and ensures integrated care, considering different devices and care levels. |
| 1. The same physician should always evaluate the same patient with oncological pain. |
| 1. The percentage of consultations to the pain unit from oncology hospitalization when the reason for admission is pain or difficult to control must be measured. |
| 1. Criteria should be established for prioritizing cancer patients to be treated in the pain units. |
| 1. A telephone follow-up plan should be established by calling the home of patients with cancer pain. |
| 1. Discomfort associated with referrals and interconsultations should be reduced (e.g., through high-intensity consultations). |
| 1. The effectiveness of the information exchange channels between the units and the professionals involved should be checked to reduce clinical variability and the possible incidence of errors associated with healthcare. |
| 1. The rehabilitation service should intervene and evaluate patients with chronic pain that is difficult to treat. |

| Training, teaching and research |
| --- |
| Criteria: |
| 1. There should be a training program on oncological pain management for healthcare personnel (doctors, nurses, clinical psychologists). |
| 1. Training stays for professionals dealing with oncological pain treatment should be valued. |
| 1. Publications should be carried out in journals included in JCR or other of contrasted quality; communications, posters to congresses; and directing doctoral theses, concerning oncological pain, evaluating the scientific activity, at least, every five years. |
| 1. The Oncology Service must participate in developing new treatments and the advances in the improvement of therapeutic effectiveness in the management of oncological pain, participating in research projects and clinical trials with direct translation to clinical practice. |
| 1. There must be a procedure to assess the patient's experience and take advantage of this information to improve the care process. |
| 1. Professional participation in courses related to bad news communication, the development of empathic understanding, and shared decision-making should be encouraged. |
| 1. Actions should be taken to prevent burnout among professionals. |

| Patient Safety |
| --- |
| 1. The occurrence of adverse events in patients with therapeutic guidelines for somatic, visceral, neuropathic and breakthrough pain should be systematically recorded and analyzed. |
| 1. An analysis of the sources of clinical variability should be carried out at appropriate intervals. |
| 1. The following agents are not recommended for early treatment or NIQT prevention: amitriptyline, IV Ca/Mg, Nimodipine, Retinoic acid, acetyl-L-carnitine, glutamine, glutathione, vitamin B6, omega-3 fatty acids, alpha-lipoic acid, acetylcysteine, and vitamin E. |
| 1. Both adverse events and near misses will be reported regularly, analyzing their causes. |
| 1. Monitoring of drug interactions that may adversely affect the patient should be carried out. |
| 1. There must be defined channels of communication between professionals for the prevention of adverse events. |
| 1. There should be guidelines for safe use of the medication. Patients should actively participate in their care and should be informed about possible errors at home related to the pain medications they have been prescribed. |
| 1. The reasons for deciding not to follow the WHO pain management ladder should be recorded in the digital medical record. |

| Patient Satisfaction |
| --- |
| 1. The assessment of the degree of dependency or autonomy available to the patient must be carried out. |
| 1. Patients and families' satisfaction with the analgesia received for the treatment of pain should be assessed, and the degree of compliance with the analgesic objectives agreed with the patient at the beginning of therapy should be evaluated. |
| 1. Patient caregiver satisfaction with the multimodal intervention received should be assessed. |
