# Supplementary material for: Pain Standards for Accredited Healthcare Organizations (ACDON Project): A Mixed Methods Study
Source: J Pers Med. 2021 Feb 5;11(2):102. doi: 10.3390/jpm11020102 (PMC7914789; doi:10.3390/jpm11020102)
Supplement: Supplementary file 1 [file jpm-11-00102-s001.zip › jpm-1063820 supplementary materials/Table S2.docx]

Table S2: Agreed quality indicators compliance and their main characteristics.

| **Area** | **No.** | **Condition** | **Standard** | **Source of information.** | **Overall compliance** |
| --- | --- | --- | --- | --- | --- |
| **1.** **Assessment and counselling for cancer pain** | 1.1 | Essential | At the time of admission, a comprehensive pain assessment should be carried out and recorded in the digital medical record in which the type and intensity of the pain described by the patient is systematically coded. | Medical record. | 87.5% |
|  | 1.2 | Recommended | To have an agreed protocol based on evidence that specifies context, assessment methods, classification, treatment, and monitoring of how pain assessment of cancer patients is carried out. | Hospital care protocol. | 87.5% |
|  | 1.3 | Recommended | Each service must have established a care route that includes adequate coordination of the patients' agenda and ensures integrated care, considering different devices and levels of care led by a multidisciplinary team. | Healthcare route document. | 75% |
|  | 1.4 | Recommended | Assess the patient's profile for appropriate use of potent opioids and substances of abuse and record it in the medical record. | Medical record. | 100% |
|  | 1.5 | Recommended | Upon admission and during patient follow-up, should be carried out a periodic reassessment, using validated scales, of the characteristics of cancer pain (e.g., NIPAC, DN4), and of its intensity and interference in basic daily activities, variables directly related to the patient's quality of life (e.g., BPI). Monitoring mean and range scores can help to measure treatment effectiveness. | Checking the medical history for the use of scales. | 75% |
| **2.** **Pharmacological treatment** | 2.1 | Essential | Patients suffering from cancer pain will have a continuous therapeutic guideline of treatment (assuring a scheduled administration, at fixed times, to avoid the onset of pain), including rescue treatment for the control of the pain crisis and breakthrough pain. | Medical record. | 100% |
|  | 2.2 | Essential | A therapeutic plan should be established by consensus with the patient (in which the patient and/or family is provided with a pharmacological treatment sheet which adequately specifies the drug prescribed and its rescue doses) which includes the analgesic objectives desired by the patient (if possible quantitative using the same initial assessment scales), as well as the temporality of its re-evaluation. | Medical record. | 100% |
|  | 2.3 | Essential | The most frequent side effects of analgesic and adjuvant treatments should be monitored. | Medical record. | 100% |
|  | 2.4 | Recommended | Preventive medication should be given for the unwanted effects of analgesics (with special consideration for the prevention and treatment of opioid-induced constipation). | Medical record. | 100% |
|  | 2.5 | Recommended | There must be a plan for monitoring the degree of therapy adherence. | Medical record. | 100% |
|  | 2.6 | Recommended | A specific record should be kept in the digital medical record of the "itinerary" through the WHO analgesic ladder, as well as the reasons for it (at the discretion of your responsible physician), to facilitate the best coordination between the responsible team and the inter-current (emergency) teams. | Medical record. | 62.5% |
| **3. Non-pharmacological treatment** | 3.1 | Essential | Psychosocial support should be offered to the patient for better pain management, from the time of diagnosis and throughout the care process according to the needs of the patient in view of the impact and repercussions of the disease on the patient and his or her social and family environment. | Medical record. | 75% |
|  | 3.2 | Recommended | A primary caregiver identification should be performed for all patients with chronic pain, along with an assessment of their degree of overload. | Medical record. | 87.5% |
|  | 3.3 | Recommended | In case of pain that does not subside with non-invasive treatment, a non-pharmacological interventional technique should be proposed to the patient. | Medical record. | 62.5% |
|  | 3.4 | Recommended | Spiritual and religious service will be offered to all those patients with chronic pain who request it. | Medical record. | 87.5% |
|  | 3.5 | Recommended | The patient should evaluate with the professional which non-pharmacological alternatives can be added to the pharmacological treatment, for example: (Encourage rest and relaxation; Diet adapted to the patient's situation. Adaptation of the physical spaces to their needs; Integration of the family in the care; Promotion of hobbies; Adaptation and flexibility in the schedules; Active attitude of the professionals; Application of cold/heat moderation; Soft massage; Aromatherapy; Reflexology; Active and passive mobilizations; Music therapy; Art therapy; Meditation; Humor and laughter therapy; Active listening; Visualization). | Medical record. | 87.5% |
| **4.** **Palliative Care** | 4.1 | Essential | All patients should have access to palliative and supportive care, throughout the course of their illness, according to the existing care structure in their geographical area. | Medical record. | 100% |
|  | 4.2 | Recommended | An agreed protocol for referral to palliative care developed from a multidisciplinary perspective should be applied. | Medical record. | 87.5% |
|  | 4.3 | Recommended | The suffering of the patient with oncological pain must be systematically assessed and accompaniment promoted. | Medical record. | 100% |
|  | 4.4 | Recommended | The patient must be informed of the basic functions of the palliative care service. | Medical record, information sheets, health education activities. | 87.5% |
| **5.Coordination** | 5.1 | Essential | Criteria for prioritizing cancer patients for care in pain units should be established. | Medical record. | 87.5% |
|  | 5.2 | Recommended | Patient information should be provided about the Pain Unit, its portfolio of services, accessibility and the strategy designed for the care of patients with chronic oncological pain in a proactive manner. | Medical record, information sheets, health education activities. | 62.5% |
|  | 5.3 | Recommended | Different services consultations should be scheduled the same day to reduce the discomfort associated with waiting lists. | High intensity visits appointment. | 50% |
|  | 5.4 | Recommended | A telephone follow-up plan should be established by calling the home of patients with cancer pain. | Medical record. | 62.5% |
| **6.** **Training, education, and research** | 6.1 | Essential | In order to guarantee equity and access to the best treatment and follow-up regardless of the patient's location, training, information and integration into the team of the doctor and nurse of the Primary Care team will be promoted to ensure maximum accessibility and continuity of care for the patient and his or her family. | Medical record. | 75% |
|  | 6.2 | Recommended | The participation of professionals in courses related to the communication of bad news, the development of empathic acuity and shared decision making should be encouraged. | Service´s continuing training record. | 87.5% |
|  | 6.3 | Recommended | Actions should be taken to prevent burnout among professionals. | Service activity log. | 25% |
|  | 6.4 | Recommended | The Pain Unit should participate in the development of new treatments and advances in the improvement of therapeutic effectiveness in the management of oncological pain, participating in research projects and clinical trials with direct translation to clinical practice. | Publications, accreditations, communications or any participation of the service in the scientific field. | 50% |
|  | 6.5 | Recommended | Training stays for professionals dealing with oncological pain treatment should be assessed. | Medical resident’s rotation record. | 75% |
| **7.** **Patient Safety** | 7.1 | Essential | The occurrence of adverse events (and near misses) in patients with therapeutic guidelines for somatic, visceral, neuropathic, breakthrough pain should be systematically recorded and analysed. | Medical record. | 87.5% |
|  | 7.2 | Recommended | There should be guidelines for safe use of the medication. Patients should actively participate in their care and should be informed about possible errors at home related to the painkillers they have been prescribed. | Medical record, information sheets, health education activities. | 100% |
|  | 7.3 | Recommended | Monitoring of drug interactions that may adversely affect the patient should be carried out. | Medical record. | 100% |
|  | 7.4 | Recommended | The adverse drug event reporting process should be used through the routes established by the AEMPS. | Adverse events recorded through the AEMPS. | 87.5% |
|  | 7.5 | Recommended | To establish effective coordination with the Hospital and Therapeutic Pharmacy Service to promote the safe use of drugs through the review of analgesia, chemotherapy and adjuvant therapy protocols, as well as to guarantee the effective dispensing of drugs both at the hospital level and in the corresponding healthcare area. | Protocol for coordinated care between services for the chronic oncology patient | 87.5% |
|  | 7.6 | Recommended | The reasons for deciding not to follow the WHO pain management ladder should be recorded in the digital medical record. | Medical record. | 62.5% |
| **8.** **Patient Satisfaction** | 8.1 | Essential | Patient and family satisfaction with the analgesia received for the treatment of pain should be assessed, as should the degree of compliance with the analgesic objectives agreed with the patient at the beginning of treatment, as well as the degree of satisfaction with the care received (personal treatment). | Service data. | 87.5% |
|  | 8.2 | Recommended | The degree of dependency or autonomy available to the patient must be assessed. | Medical record. | 100% |
|  | 8.3 | Recommended | Caregiver satisfaction with the multimodal intervention received should be assessed. | Service data. | 75% |
|  | 8.4 | Recommended | There must be a procedure for assessing the patient's experience and using this information to improve the care process. | Service data. | 50% |
